# *SFRP4* gene expression is increased in aggressive prostate cancer

**DOI:** 10.1038/s41598-017-14622-3

**Published:** 2017-10-27

**Authors:** Elise Sandsmark, Maria K. Andersen, Anna M. Bofin, Helena Bertilsson, Finn Drabløs, Tone F. Bathen, Morten B. Rye, May-Britt Tessem

**Affiliations:** 1 Department of Circulation and Medical Imaging, Faculty of Medicine and Health Sciences, NTNU - Norwegian University of Science and Technology, Trondheim, Norway; 2 Department of Clinical and Molecular Medicine, Faculty of Medicine and Health Sciences, NTNU - Norwegian University of Science and Technology, Trondheim, Norway; 3 Department of Urology, St. Olav’s Hospital, Trondheim University Hospital, Trondheim, Norway; 4 Clinic of Surgery, St. Olav’s Hospital, Trondheim University Hospital, Trondheim, Norway

## Abstract

Increased knowledge of the molecular differences between indolent and aggressive prostate cancer is needed for improved risk stratification and treatment selection. Secreted frizzled-related protein 4 (SFRP4) is a modulator of the cancer-associated Wnt pathway, and previously suggested as a potential marker for prostate cancer aggressiveness. In this study, we investigated and validated the association between *SFRP4* gene expression and aggressiveness in nine independent cohorts (n = 2157). By differential expression and combined meta-analysis of all cohorts, we detected significantly higher *SFRP4* expression in cancer compared with normal samples, and in high (3–5) compared with low (1–2) Grade Group samples. *SFRP4* expression was a significant predictor of biochemical recurrence in six of seven cohorts and in the overall analysis, and was a significant predictor of metastatic event in one cohort. In our study cohort, where metabolic information was available, *SFRP4* expression correlated significantly with the concentrations of citrate and spermine, two previously suggested biomarkers for aggressive prostate cancer. SFRP4 immunohistochemistry in an independent cohort (n = 33) was not associated with aggressiveness. To conclude, high *SFRP4* gene expression is associated with high Grade Group and recurrent prostate cancer after surgery. Future studies investigating the mechanistic and clinical usefulness of *SFRP4* in prostate cancer are warranted.

## Introduction

Prostate cancer is the second most common cancer and the fifth leading cause of cancer related death in men worldwide^1^. The lack of accurate markers to separate aggressive from non-aggressive prostate cancer at an early time point is causing considerable overtreatment of indolent cancers^2^. Discovery of new biomarkers of aggressiveness, as well as improved understanding of differences between indolent and aggressive prostate cancer, are therefore highly needed.

The family of secreted frizzled-related proteins (SFRP1–5) are extracellular inhibitors of Wnt signalling, a pathway identified for its role in carcinogenesis^3^. The SFRPs are in general regarded as tumour suppressors, however, oncogenic properties have also been suggested due to biphasic modulation of Wnt signalling^4,5^ and interactions with other signalling pathways^4^. SFRP4 is the largest and the most structurally different of the family members^6^. In several types of cancer, SFRP4 follows a tumour suppressor pattern with epigenetic silencing and reduced gene expression, as reviewed by Pohl *et al*.^7^. However, for prostate cancer, increased gene expression of *SFRP4* has been observed^8,9^, and shown to be a predictor of recurrent disease^10^. Additionally, *SFRP4* has been included in different gene expression signatures linked to prostate cancer aggressiveness and recurrence^10,11^, including our previously published signature for non-canonical Wnt pathway and epithelial-to-mesenchymal transition (NCWP-EMT) markers^12^. Protein levels of SFRP4 measured by immunohistochemistry is discordant in prostate cancer; Horvath *et al*.^13,14^ reported increased expression of membranous SFRP4 staining to be associated with good prognosis, while Mortensen *et al*.^10^ reported cytoplasmic expression to be linked to worse prognosis. Overall SFRP4 appears to be a potential biomarker candidate for prostate cancer aggressiveness, and there is a need to validate and clarify the role of SFRP4 in prostate cancer.

Reprogramming of metabolism is one of the hallmarks of cancer development^15^. For prostate cancer, the metabolites citrate and spermine have shown promise as biomarkers and are found in lower concentrations in aggressive compared to indolent cancers^16,17^. Our NCWP-EMT gene expression signature was associated with reduced concentrations of these metabolites^12^, but the correlation between *SFRP4* gene and protein expression levels, and citrate and spermine has not previously been investigated in prostate cancer. Our previously published method for integration of gene expression levels with metabolic data and histopathology of the exact same samples, gives an excellent opportunity to examine this^18^.

The overall aim of this study was to investigate and validate *SFRP4* gene expression in prostate cancer, and its relation to cancer aggressiveness. The results were validated in eight independent, publically available gene expression prostate cancer cohorts with patient follow-up data. Furthermore, SFRP4 protein expression was assessed using immunohistochemistry in a separate cohort. Our approach of including several independent patient cohorts gave increased statistical power, and improved the accuracy and generalisation of the results.

## Results

Our study cohort consisted of 156 prostate tissue samples from 41 patients, of which 116 were cancer tissue samples^19^. Eight independent prostate cancer validation cohorts were downloaded from Gene Expression Omnibus (GEO) and The Cancer Genome Atlas (TCGA), giving a total number of 2157 samples from 1884 patients. Five of the validation cohorts included normal samples as well as cancer samples. Our additional patient cohort for immunohistochemistry analysis, termed the IHC cohort^12^, included prostate cancer samples from 40 patients. Clinical and histopathological data for all patient cohorts included in the study are listed in Table 1.

**Table 1:** Clinical and histopathological variables of all ten cohorts.

Clinical variables	Study cohort	IHC cohort	Erho *et al*.	TCGA-PRAD	CAM Ross-Adams *et al*.
Samples (patients)	156 (41)	40 (40)	545 (545)	549 (497)	186 (163)
Cancer samples (patients)	116 (41)	40 (40)	545 (545)	497 (497)	112 (112)
Age at diagnosis, years (median, range)	64 (48–69)	61 (48–73)	65.3 ± 6.4	61 (41–78)	61 (41–73)
PSA before surgery, ng/mL (median, range)	9.1 (4.0–45.8)	8.85 (5.2–18)	—	7.4 (0.7–107)	7.8 (3.2–23.7)
Grade Groups					
Low (1–2)	60 (52%)	19 (47.5%)	334 (61%)^a^	207 (42%)	82 (73%)
High (3–5)	56 (48%)	21 (52.5%)	211 (39%)^a^	289 (58%)	30 (27%)
Pathological T stage					
pT1	—	—	—	—	—
pT2	70 (60%)	27 (68%)	219 (40%)	187 (38%)	33 (29%)
pT3	40 (35%)	12 (30%)	253 (47%)	293 (59%)	74 (66%)
pT4	—	—		9 (2%)	1 (1%)
No data	6 (5%)	1 (2%)	73 (13%)	8 (1%)	4 (4%)
Follow-up					
Endpoint	BCR	BCR	Metastasis	BCR	Recurrence
Occurred	13 (32%)	16 (40%)	212 (39%)^b^	91 (18%)	19 (17%)
Not occurred	21 (51%)	21 (53%)	333 (69%)^b^	399 (80%)	93 (83%)
No data	7 (17%)	3 (8%)	—	7 (2%)	—
**Clinical variable**	**STK Ross-Adams** ***et al***.	**Wang** ***et al***.	**Sboner** ***et al***.	**Taylor** ***et al***.	**Mortensen** ***et al***.
Samples (patients)	94 (94)	136 (82)	281 (281)	160 (131)	50 (50)
Cancer samples (patients)	94 (94)	65 (56)	281 (281)	131 (131)	36 (36)
Age years (median, range)		63 (43–77)	74 (51–91)	58 (37–73)	63 (46–71)
PSA before surgery, ng/mL (median, range)	7.95 (1.5–117)		6.62 (1.0–75)	5.92 (1.0–46)	16 (5.0–43)
Grade Groups					
Low (1–2)	60 (64%)	50 (77%)	162 (58%)	107 (82%)	32 (89%)^a^
High (3–5)	34 (36%)	15 (23%)	119 (42%)	24 (18%)	4 (11%)^a^
Pathological T-stage					
pT1	—	1 (2%)	281^c^ (100%)	—	—
pT2	48 (51%)	32 (57%)	—	85 (65%)	19 (53%)
pT3	42 (45%)	20 (35%)	—	40 (30%)	17 (47)
pT4	—	1 (2%)	—	6 (5%)	—
No data	4 (4%)	2 (2%)	—	—	—
Follow-up					
Endpoint	Recurrence	BCR	PCa-death	BCR	BCR
Occurred	45 (48%)	29 (52%)	165 (59%)	27 (21%)	22 (61%)
Not occurred	48 (51%)	27 (48%)	116 (41%)	104 (79%)	14(39%)
No data	1 (1%)	—	—	—	—

Abbreviations: BCR – biochemical recurrence, PCa-death – prostate cancer-specific death.

^a^In Erho *et al*. and Mortensen *et al*.: Low Grade Group 1–3 and high Grade Group 4–5 (due to lack of information to separate Grade Group 2 and 3).

^b^In Erho *et al*. metastatic progression at 10-year patient follow-up.

^c^Clinical T-stage.

### *SFRP4* expression in cancer

**Figure 1:**
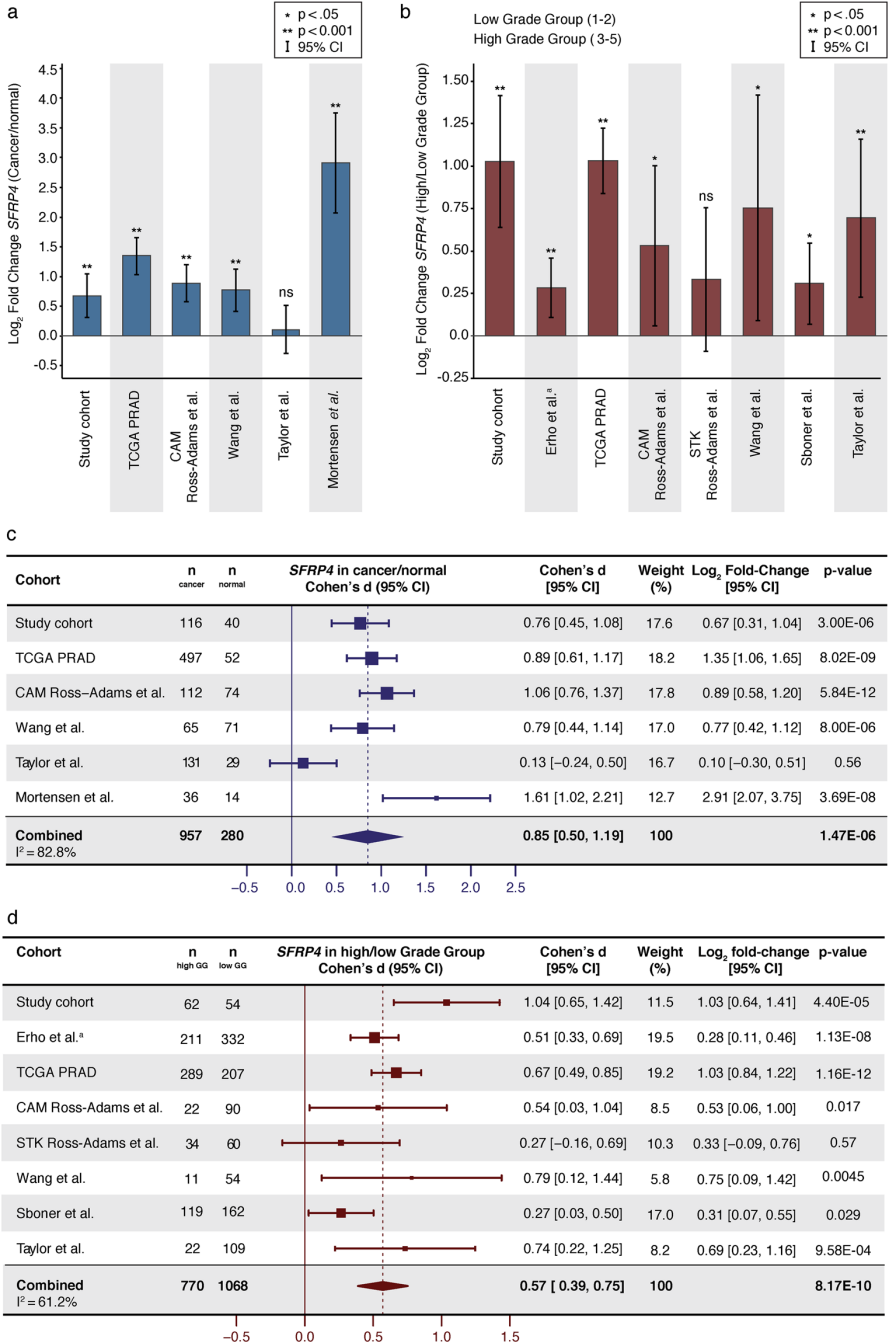
*SFRP4* gene expression in prostate cancer. (**a**) Log_2_ fold change of *SFRP4* expression in cancer compared with normal samples (**b**) Log_2_ fold change of *SFRP4* expression in high Grade Group (3–5) compared with low Grade Group (1–2) samples. (**c**) Forest plot and meta-analysis of *SFRP4* expression in prostate cancer compared with normal prostate samples. (**d**) Forest plot and meta-analysis of *SFRP4* expression in high Grade Group compared with low Grade Group prostate cancer samples. Abbreviations: ns - not significant, GG – Grade Group, CI – confidence interval. ^a^In the Erho *et al*. cohort, low Grade Group included GG 1–3, and high Grade Group included GG 4–5.

In our study cohort, there was significantly higher *SFRP4* expression in cancer samples compared with normal samples (t-test p < 0.001, Fig. 1a). This was also true for four of the five independent validation cohorts which included expression data from both cancer and normal samples (Fig. 1a). Meta-analysis of all the cohorts gave a significant combined Cohen’s *d* of 0.85 (p < 0.001, Fig. 1c). This is considered a large effect-size and indicates a considerable difference in mean *SRFP4* expression between normal and cancer tissue. Together, this clearly shows significant upregulation of *SFRP4* in prostate cancer compared with normal prostate tissue.

### *SFRP4* expression in cancer with high Grade Group

In our study cohort, there was significantly higher *SFRP4* expression in high Grade Groups (3–5) compared with low Grade Groups (1–2) cancer samples (t-test p < 0.001, Fig. 1b), and this was confirmed in six of the seven validation cohorts (Fig. 1b). Meta-analysis of all the analysed cohorts further strengthened this finding, giving a significant combined Cohen’s *d* of 0.57 (p < 0.001, Fig. 1d). The Mortensen *et al*. cohort was excluded from differential expression analysis between high and low Grade Groups due to the low number of high Grade Group samples (n = 4).

### *SFRP4* expression and pathological T-stage and preoperative PSA value

*SFRP4* expression was significantly higher in samples from patients with a high pathological T-stage (≥T3a) compared with low T-stage samples (≤T2c) in six out of the seven cohorts that included information on T-stage (Supplementary Table S1). *SFRP4* expression was not correlated with preoperative PSA in any of the cohorts (Supplementary Table S1).

### *SFRP4* and patient follow-up

**Figure 2:**
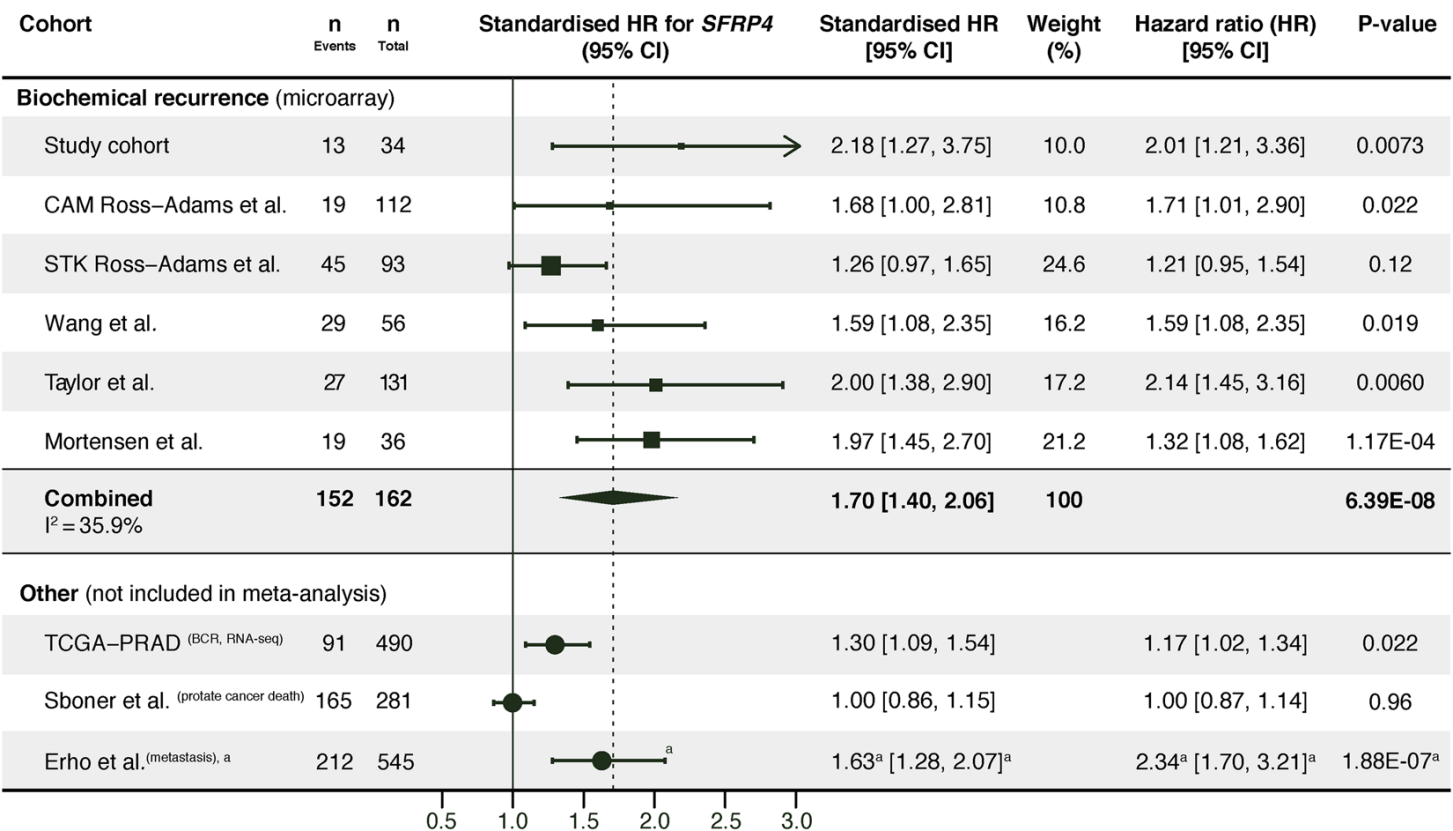
Univariate Cox proportional hazard analysis of *SFRP4* expression and follow-up endpoints. *SFRP4* gene expression was used as a continuous variable in the analyses. Meta-analysis was performed on the cohorts with microarray based *SFRP4* gene expression data and biochemical recurrence (PSA ≥ 0.2 ng/mL) as endpoint. For the cohorts with multiple samples per patients (study and Wang *et al*. cohort), one sample per patient was selected by random. Abbreviations: CI – confidence interval, HR – hazard ratio, BCR – biochemical recurrence. ^a^The Erho *et al*. cohort was analysed by logistic regression, with odds ratio as the effect size.

In our study cohort, the continuous value of *SFRP4* expression was a significant predictor of biochemical recurrence (PSA ≥ 0.2 ng/mL) after radical prostatectomy by univariate Cox proportional hazards analysis (p = 0.007, Fig. 2). This was further confirmed in five of the six validation cohorts with biochemical recurrence as endpoints (Fig. 2). Meta-analysis of the six cohorts with microarray based gene expression gave a significant combined *SFRP4* standardised hazard ratio (HR) of 1.70 for prediction of biochemical recurrence (p < 0.001, Fig. 2). Continuous *SFRP4* expression was not a predictor of prostate cancer-specific death in the watchful waiting Sboner *et al*. cohort (HR 1.0, p = 0.96, Fig. 2). Logistic regression analysis showed *SFRP4* expression to be a predictor of metastases after radical prostatectomy in the Erho *et al*. cohort (odds ratio 2.34, p < 0.001, Fig. 2).

### *SFRP4* expression and metabolism

**Figure 3:**
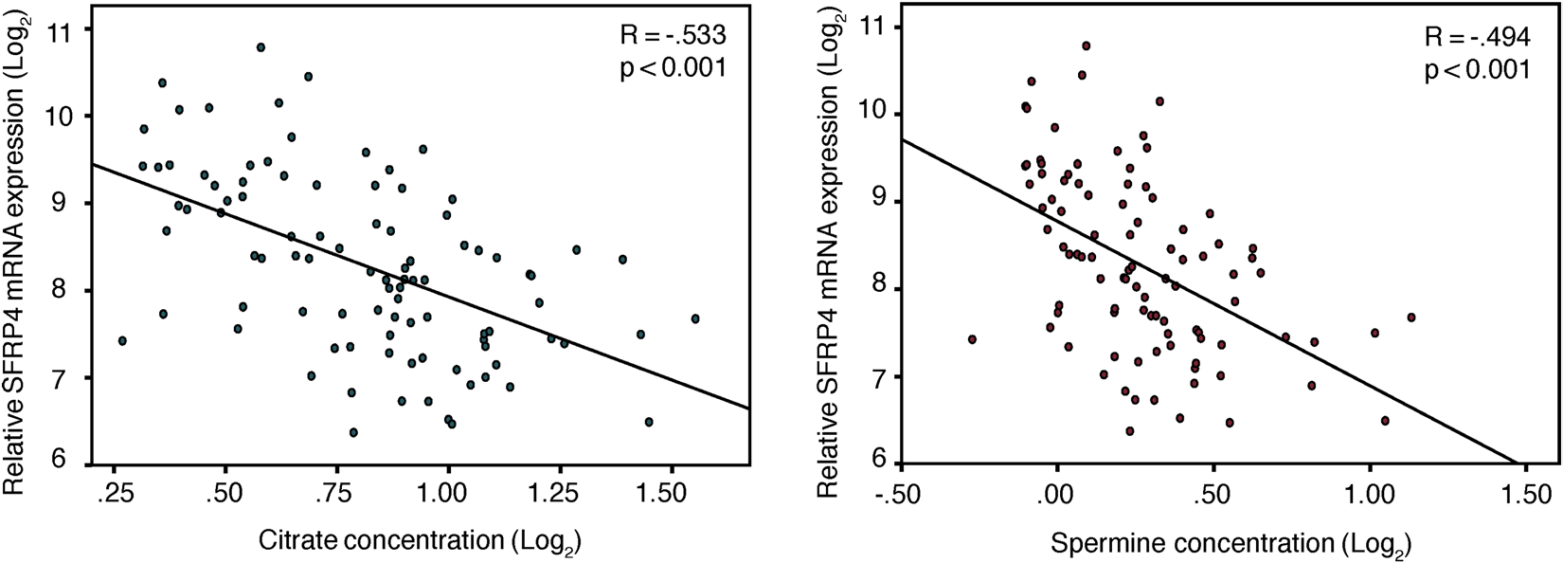
Correlations with metabolism. Linear Pearson correlations between *SFRP4* gene expression and citrate and spermine in our study cohort. All variables are log_2_ transformed.

In our study cohort, the *SFRP4* expression level was negatively correlated with concentrations of citrate (Pearson’s r = −0.53, p < 0.001) and the polyamine spermine (Pearson’s r = −0.49, p < 0.001) (Fig. 3). These were the strongest correlations to citrate and spermine of all the genes in our previously published NCWP-EMT gene expression signature^12^ (Supplementary Table S2).

### SFRP4 immunohistochemistry

**Figure 4:**
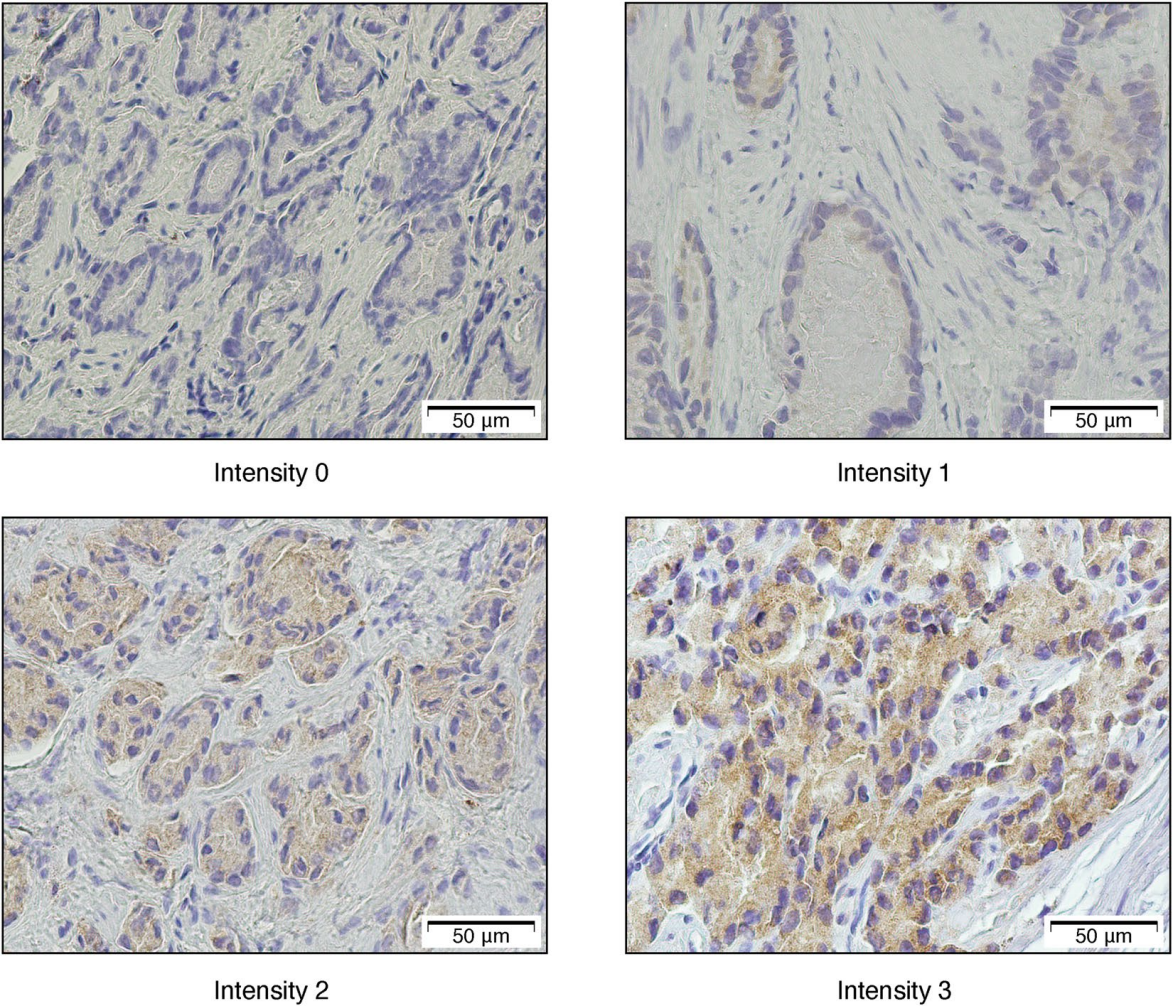
Immunohistochemistry of SFRP4. Examples of staining intensities 0 to 3 in our IHC cohort.

In our IHC cohort, seven of the 40 samples were excluded from further analysis due to insufficient or lack of tumour cells in the immunohistochemically stained sections. We did not detect membranous SFRP4 staining of prostate cancer cells in any samples. However, cytoplasmic SFRP4 staining of different intensities was identified and categorised into four different scores (Fig. 4). Proportion of positive cancer cells were also scored and multiplied with staining intensity to create a staining index (Supplementary Table S3). Full immunohistochemistry scoring of each sample along with clinical, histopathological and metabolic data is shown in Supplementary Table S4.

There was no relationship between Grade Groups and SFRP4 staining index (Fisher’s exact p = 1.0). This was also the case when looking at staining intensity and proportion separately (Fisher’s exact p = 0.80 and p = 0.82, respectively). Furthermore, no significant associations between SFRP4 staining and biochemical recurrence (Log-rank: staining index p = 0.87, intensity p = 0.82, proportion p = 0.95), nor any significant correlation between SFRP4 staining index and citrate and spermine concentrations (Pearson’s r = 0.13 p = 0.47 and Pearson’s r = 0.18 p = 0.32, respectively) were detected.

## Discussion

In this study, we performed analyses of *SFRP4* gene expression and validated the results in eight independent prostate cancer cohorts. We showed *SFRP4* expression to be increased in prostate cancer, and further increased in high Grade Group (3–5) compared with low Grade Group (1–2) cancers. Additionally, *SFRP4* gene expression was found to be a predictor of worse outcome in prostatectomy-treated prostate cancer patients. Furthermore, the *SFRP4* expression level was negatively correlated with the concentrations of citrate and spermine in the samples. Together, these results underpin *SFRP4* as a biomarker candidate of prostate cancer aggressiveness.

We showed *SFRP4* gene expression to be increased in prostate cancer compared with normal tissue in five of six cohorts, and in the combined meta-analysis of all cohorts. This is in agreement with Luo *et al*.^8^ and Wissmann *et al*.^9^, who investigated matched tumour and normal tissue samples from 16 and 56 prostate cancer patients, respectively. Contradictory, García-Tobilla *et al*.^20^ did not find significantly different expression levels of *SFRP4* between normal and prostate cancer tissue, however, this study suffered from small sample size (normal n = 4, cancer n = 11). In a previous paper, we also showed increased *SFRP4* expression in prostate cancer when balancing for stroma content in the samples^12^. Interestingly, two studies have shown increased *SFRP4* expression in prostate cancer tissue compared with benign prostate hyperplasia^20,21^, but this approach was not possible to pursue in our study. To summarise, previous studies have in general reported increased *SFRP4* gene expression in prostate cancer compared with normal prostate tissue, but they have been carried out on small cohorts. The result of the present study adds substantial validation to *SFRP4* expression being increased in prostate cancer.

We showed increased expression of *SFRP4* in high Grade Group compared with low Grade Group cancer samples, as well as an association between *SFRP4* expression and risk of biochemical recurrence and metastasis after radical prostatectomy. *SFRP4* gene expression has previously been linked to more aggressive prostate cancer; Luo *et al*.^8^ showed increased expression of *SFRP4* in tissue samples from prostate cancer patients with pathological stage T3a-b compared with pathological stage T2b. Mortensen *et al*.^10^ discovered *SFRP4* to be a part of two aggressive gene expression clusters, as well as being an independent predictor of recurrence after prostatectomy in an independent cohort. Our previously published NCWP-EMT gene expression signature, which included *SFRP4* as one of 15 genes, was associated with biochemical recurrence and metastasis after prostatectomy^12^. Furthermore, Oncotype DX^®^ Prostate Cancer, a commercially available gene expression signature, includes *SFRP4* as one of its 17 genes, and has been associated with clinical recurrence of prostate cancer after prostatectomy^11^. Our analyses of multiple independent cohorts in the current study, further support high *SFRP4* expression to be associated with more aggressive prostate cancer. To conclude, several studies^8,10–12^, including the current study, support *SFRP4* gene expression to be upregulated in aggressive compared with less aggressive prostate cancer.

SFRP4 is classified as an inhibitor of Wnt signalling, a pathway implicated in carcinogenesis^3^. Consequently, SFRP4 is expected to be a tumour suppressor, and to be downregulated in aggressive cancer. As reviewed by Pohl *et al*., DNA hypermethylation of the *SFRP4* promotor and reduced *SFRP4* gene expression is observed in many types of cancers, including endometrial, ovarian, bladder, and oesophageal cancer^7^. Although *SFRP4* expression in prostate cancer tissue seems to deviate from this, a few prostate cell line studies have supported tumour suppressor properties of SFRP4 also in prostate cancer; Horvath *et al*.^13,14^ showed that PC3 and LNCaP cell lines modified to overproduce SFRP4 proteins had reduced cellular proliferation compared to controls. Furthermore, García-Tobilla *et al*.^20^ showed reduced gene expression of *SFRP4* in prostate cancer cell lines (LNCaP, PC3, DU145 and 22Rv1) compared with control cells (PREC). However, they did not detect DNA hypermethylation at the *SFRP4* promotors that could explain this downregulation in any of the cell lines^20^. Absence of *SFRP4* hypermethylation was also shown in both prostate cancer cell lines and in tumour tissue in a study by Perry *et al*.^21^. In contrast to the study by García-Tobilla *et al*.^20^, and in coherence with human prostate cancer tissue studies, Perry *et al*.^21^ also detected upregulation of *SFRP4* in all prostate cancer cell lines (LNCaP, PC3, DU145 and 22Rv1) compared with controls (PWR-1 and RWPE1). Interestingly, in the two latter mentioned studies^20,21^, DNA hypermethylation of *SFRP2*, *SFRP3*, and *SFRP5* was detected in both cell lines and human prostate cancer tissues^20,21^. This is in agreement with findings in colorectal cancer, where Suzuki *et al*.^22^ suggested that SFRP4 may not be an important inhibitor of the Wnt signalling pathway due to lower frequency of DNA hypermethylation and weaker Wnt signalling inhibition compared with other SFRP family members. This may be translatable to prostate cancer, and could explain why *SFRP4* is not downregulated in cancer. However, studies of how SFRP4 regulates the Wnt signalling pathway and other pathways in prostate cancer are necessary before a conclusion can be drawn.

In the current study, we detected an association between *SFRP4* expression and development of metastases after prostatectomy in the Erho *et al*. cohort. Bones are the most frequent site for haematogenous metastases in prostate cancer^23^, and, interestingly, SFRP4 has been suggested to have an important role in bone homeostasis^24,25^. However, to our knowledge, the function of SFRP4 in bone metastases has not been investigated. A hypothesis to explain the association between *SFRP4* gene expression and high Grade Groups, as well as recurrence and metastasis after prostatectomy, could therefore be that SFRP4 increases the cancer cell’s ability to metastasise to bone. Future studies investigating the role of SFRP4 in prostate cancer bone metastases would consequently be of interest.

For patient follow-up in this study, we used the surrogate endpoints of biochemical recurrence and metastases, in all except one cohort, Sboner *et al*., in which prostate cancer-specific death was used. Such surrogate endpoints are commonly used in prostate cancer studies, due to a natural long survival time of patients. Although recurrence after prostatectomy in general represents more aggressive disease, we also recognise that the use of surrogate endpoints is a limitation to the clinical value of the results, as only a minority of patients with biochemical recurrence will experience systemic progression or prostate cancer-specific death^26^. In the Sboner *et al*. cohort, we did not see any association between *SFRP4* gene expression and cancer-specific death. This cohort did, however, differ substantially from the other analysed cohorts, where all patients included had incidental prostate cancer discovered by trans-urethral resection of the prostate (TURP), and were classified as stage T1a-T1b, NX, and M0 disease. The samples used for gene expression were from the TURP procedure. Although most prostate cancers arise from the peripheral zone, resection performed by TURP represents the transition zone, and is likely to detect a higher rate of transition zone prostate cancers. Substantial differences in gene expression between tumours of different zonal origin has previously been observed^27^. This may limit future clinical use of *SFRP4* expression for risk stratification in patients with transitional zone prostate cancers, and potentially also in patients with very early stage prostate cancer, and this should be further investigated.

Changes in metabolism is regarded as one of the hallmarks of cancer^15^. In prostate cancer, the concentrations of the metabolites citrate and spermine are shown to be reduced in cancer compared with normal tissue^28,29^, and further reduced in high Gleason score prostate cancer^16^. A recent study has also shown citrate and spermine to be predictors of prostate cancer biochemical recurrence in three independent cohorts^17^. The high negative correlation between *SFRP4* expression and spermine and citrate in our study cohort further supports *SFRP4* expression to be associated with aggressive cancer. One of the normal functions of prostate cells is production of citrate and spermine for the prostatic fluid, and reduced concentration of these metabolites may signify loss of normal prostate function. However, whether these metabolic mechanisms are directly related to *SFRP4* expression was not investigated in the current study.

We did not find any association between immunohistochemical staining of SFRP4 and histopathological, metabolic, or follow-up data in our IHC cohort in this study. Our cohort only included tissue samples from 33 patients, as it was originally part of a demanding integrated analysis of metabolomics, histopathology, and patient follow-up^12,30^. This small sample size limits the interpretation of our immunohistochemistry results. There are only four previous studies including immunohistochemistry of SFRP4 in prostate cancer, and there are no standardised protocols for staining or scoring. Three of these studies were based on the same SFRP4 stained cohort of tissue microarray (TMA) samples from 229 radical prostatectomy patients^13,14,31^, where membranous SFRP4 staining was found to be associated with good prognosis^13^. In the current study, we did not observe any membranous staining of SFRP4. The lack of membranous staining is in accordance with a previous study by Mortensen *et al*.^10^, which included TMA sections from 517 radical prostatectomy patients. Our IHC cohort was stained by the same commercially available antibody and in the same dilution as used in the Mortensen *et al*. study^10^, which may explain the similar staining pattern. The use of different antibodies compared with the Horvath *et al*. study^13^ may be a possible cause of the observed disparity in membranous staining. In addition, the relatively weak staining of SFRP4 in the current study (Fig. 4) may have been insufficient to demonstrate membrane staining. In contrast to the TMA sections used in both the Mortensen *et al*.^10^ and Horvath *et al*.^13^ studies, our IHC cohort consisted of sections from needle biopsy samples. These biopsy sections were larger than the standard TMA sections, and we observed staining intensity heterogeneity within each sample. Consequently, we experienced some challenges when determining the immunohistochemistry intensity score of the samples. As mentioned, there are limitations of the immunohistochemistry evaluation of SFRP4 in the current study, especially with regard to the small sample size, and as a consequence, no certain conclusion can be made based on our results. Nevertheless, we have demonstrated a few issues that are important to address before immunohistochemistry of SFRP4 can have a role in prostate cancer risk stratification, including the lack of standardised staining and evaluation protocols, and the uncertain impact of staining heterogeneity.

In the current study, we did not directly investigate possible clinical applications of *SFRP4* expression, and this should be examined in future studies. Absolute quantification of *SFRP4* mRNA by real time PCR in biopsies may have a role for risk stratification and treatment selection for prostate cancer patients, including selection of patients for active surveillance and adjuvant treatment. Another interesting possibility for further studies, is investigation of the *SFRP4* gene and protein expression levels in less invasive liquid biopsies such as serum, urine, and prostatic and seminal fluid.

In this study, we have validated the presence of increased *SFRP4* gene expression in prostate cancer tissue, and we detected and validated higher *SFRP4* gene expression in high Grade Group compared with low Grade Group cancer. We further showed that the *SFRP4* gene expression level was as a predictor of recurrence and metastasis after prostatectomy. Finally, we showed a negative correlation between *SFRP4* gene expression and the indolent metabolic markers, citrate and spermine. To conclude, *SFRP4* expression is associated with more aggressive disease, and is a biomarker candidate for risk stratification of prostate cancer patients. *SFRP4* may therefore potentially be useful in the selection of candidates for active surveillance as well as for patients in need of adjuvant or more aggressive treatment, and *SFRP4* deserves further attention in prostate cancer studies.

## Methods

### Ethics statement

The study was approved by and carried out in accordance to the regulations of the Regional Committee for Medical and Health Research Ethics, Central Norway (identifiers 4.2007.1890 and 4.2007.1654). All patients signed a written informed consent.

### Patients and samples

The samples in our study and IHC cohort were donated from patients diagnosed with localised or locally advanced prostate cancer and treated with radical prostatectomy between 2007 to 2010 at St. Olav’s Hospital, Trondheim University Hospital. None of the patients had received any prostate cancer treatment prior to surgery. Samples in the study cohort were harvested from fresh-frozen prostatectomy specimens with a highly standardised method as previously described by Bertilsson *et al*.^18^. The samples in the IHC cohort were collected as needle biopsies within approximately two minutes after the prostatectomy, and were snap frozen.

### Follow-up

At least five-year follow-up data were collected for the patients in our study and IHC cohort. Biochemical recurrence was defined as serum PSA levels of at least 0.2 ng/mL in two independent measurements, or, in case of missing PSA values/time-point, onset of salvage therapy.

### Histopathology

For histopathological evaluation, a cryosection from each fresh frozen tissue sample in our study cohort and two formalin-fixed paraffin-embedded sections of each sample in our IHC cohort were used. All sections were evaluated by an experienced pathologist as previously described^12^. The reproducibility of the histopathological evaluation has previously been assessed in our study cohort, by an independent pathologist blinded for previous evaluation, where high interrater agreement (κ = 0.66) was reported^12^. Post-operative Gleason score was obtained from the whole-mount prostate sections according to the clinical criteria for prostate cancer. The reported Gleason scores were directly converted to Grade Groups according to the new grading system for prostate cancer^32^. For statistical analyses, the samples and patients were divided into two groups: low Grade Group (1–2) and high Grade Group (3–5).

### Metabolomics

The samples in the study cohort and IHC cohort were analysed by proton high-resolution magic angle spinning magnetic resonance spectroscopy (HR-MAS MRS) using a Bruker Avance DRX600 Spectrometer (Bruker Biopsin, Germany). LCModel was applied for absolute quantification of 23 metabolites from the spectra. More details on the HR-MAS MRS acquisition and metabolite quantification have been described by Giskeødegård *et al*. for the study cohort^16^ and Hansen *et al*. for the IHC cohort^30^.

### Microarray gene expression

Gene expression analysis was performed on the tissue samples in the study cohort after HR-MAS MRS. Illumina Human HT-12v4 Expression Bead Chip (Illumina) were used to measure relative gene expression as previously described by Bertilsson *et al*.^19^.

### Immunohistochemistry

In the IHC cohort, immunohistochemistry was performed using 4 μm thick, formalin fixed, paraffin embedded tissue sections. Rabbit polyclonal antibody against SFRP4 (Protein Tech catalogue: 15328-1-AP) was used in a 1:200 dilution at pH 9. The sections were counterstained with haematoxylin. Every section was evaluated for SFRP4 staining location (membranous or cytoplasmic). The most common staining intensity of each sample was scored from 0 to 3 (Fig. 4) based on the staining intensities described by Mortensen *et al*.^10^. Additionally, the proportion of positive cancer cells was scored from 0 to 3, and was multiplied by the intensity score to obtain a staining index (0–9). For statistical analyses, the staining index was categorised into three groups (0, 1–3, and 4–9). Further details on the scoring procedure are given in Supplementary Table S3. Two independent readings were performed by one pathologist experienced in immunohistochemistry and one physician. When scoring differed, consensus was reached by re-evaluation of the sections together.

### Validation cohorts

For validation, the following seven prostate cancer cohorts with available microarray gene expression and follow-up data were downloaded from GEO: Erho *et al*. (GSE46691)^33,34^, CAM (Cambridge) Ross-Adams *et al*. (GSE70768)^35^, STK (Stockholm) Ross-Adams *et al*. (GSE70769)^35^, Wang *et al*. (GSE8218)^36–38^, Sboner *et al*. (GSE16560)^39^, Taylor *et al*. (GSE21035/32)^40^, and Mortensen *et al*. (GSE46602)^10^. In addition, a RNA sequencing (RNA Seq) cohort of prostate adenocarcinomas, TCGA PRAD, was downloaded from TCGA^41,42^. Cancer samples for all cohorts were from radical prostatectomy specimens, except Sboner *et al*. which was from a watchful waiting patient cohort of incidental prostate cancer discovered by transurethral resection of the prostate. Normal samples in Mortensen *et al*. were from surgical prostate specimens from patients with bladder cancer, four of the normal prostate samples in Wang *et al*. were autopsy samples from normal subjects, the rest and the other cohorts were adjacent normal prostate tissue from prostatectomy specimens. According to histopathology, the samples were divided into the same two groups as our cohorts: low Grade Group (1–2) and high Grade Group (3–5). The Erho *et al*. and Mortensen *et al*. cohorts did not included information to separate Grade Group 2 and 3, and for these cohorts the low and high Grade Groups were defined as Grade Group 1–3 and 4–5, respectively. Biochemical recurrence was the follow-up endpoint in Wang *et al*., Taylor *et al*., Mortensen *et al*., and TCGA PRAD. Biochemical recurrence and/or salvage treatment were the recurrence endpoints in the CAM and STK Ross-Adams *et al*. cohorts. For the Erho *et al*. cohort, metastasis was the endpoint, and prostate cancer-specific death was the endpoint in the Sboner *et al*. cohort. Clinical and histopathological data of the cohorts are listed in Table 1, and an overview table of the cohorts is included as Supplementary Table S5.

### Statistical analysis

When more than one probe for *SFRP4* existed in a cohort, the probe with the highest variance was chosen for statistical analyses. For all analyses, *SFRP4* gene expression data were log_2_ transformed if not previously performed. For the gene expression cohorts, independent sample t-tests (two-tailed) were used for comparisons between two groups. Q-Q plots were used to check the normality assumption; small deviations were accepted due to the robustness of the test. Equal variance assumption was tested by Levene’s test, and corrected for when applicable. Fieller’s method was used to obtain pooled confidence interval for the log_2_ fold changes. To obtain Cohen’s *d*, a standardised effect size for meta-analyses, the difference between two means (cancer and normal, and high and low Grade Group) were divided by their pooled standard deviation. Meta-analyses by random-effect model were performed using the *metafor* package^43^ in R^44^.

In the two cohorts with multiple samples per patients (our study cohort and the Wang *et al*. cohort), one sample per patient was randomly selected for survival analyses. Univariate Cox proportional hazard regression analyses were performed on the continuous *SFRP4* expression. The proportional hazard assumption was tested using the *survival* package^45^ in R^44^. Standardised hazard ratios were obtained by multiplying the natural logarithm of the hazard ratio (beta) by its standard deviation^46^. Cohorts with microarray based gene expression data and biochemical recurrence as endpoint were included in a random-effect model meta-analysis, which was performed in R^44^ using the *metafor* package^43^. Due to unavailable time-point of the events in the Erho *et al*. cohort, logistical regression was used for the follow-up analyses of this cohort.

A two-tailed t-test was performed on *SFRP4* expression between high pathological T-stage (≥T3a) and low T-stage (≤T2c) for the seven cohorts that included information about T-stage. The two cohorts excluded from this analysis were the Erho *et al*. cohort where T-stage information was only available for the whole cohort and not for the individual samples, and the Sboner *et al*. cohort which only included clinical T-stage (all T1c). Pearson correlation was performed to calculate the correlation coefficient (Pearson’s r) between preoperative PSA and *SFRP4* expression for the same cohorts.

Pearson correlation coefficients (two-tailed) were used to test the correlations between gene expression and log_2_ transformed concentrations of the metabolites citrate and spermine in our study and IHC cohort. Fisher exact tests (two-tailed) were used to examine the relationship between immunohistochemistry staining and histopathological Grade Groups, and log-rank statistics were used to investigate the relationship between SFRP4 staining and time to biochemical recurrence.

For all statistical tests the significant level was set at p = 0.05. When mentioned, analyses were performed in R^44^, all other analyses were performed in SPSS^47^.

## Electronic supplementary material

Supplementary Information
