# Supplementary material for: SFRP4 gene expression is increased in aggressive prostate cancer
Source: Sci Rep. 2017 Oct 27;7:14276. doi: 10.1038/s41598-017-14622-3 (PMC5660209; doi:10.1038/s41598-017-14622-3)
Supplement: Supplementary file 1 — Supplementary Information [file 41598_2017_14622_MOESM1_ESM.doc]

## Supplementary to:

## *SFRP4* gene expression is increased in aggressive prostate cancer

Elise Sandsmark^1^*, Maria K. Andersen^1^, Anna M. Bofin^2^, Helena Bertilsson^3,4^, Finn Drabløs^4^, Tone F. Bathen^1^, Morten B. Rye^4,5Δ^, May-Britt Tessem^1Δ^*

1. Department of Circulation and Medical Imaging, Faculty of Medicine and Health Sciences, NTNU - Norwegian University of Science and Technology, Trondheim, Norway
2. Department of Laboratory Medicine, Children’s and Women’s Health, Faculty of Medicine and Health Sciences, NTNU - Norwegian University of Science and Technology, Trondheim, Norway
3. Department of Urology, St. Olav’s Hospital, Trondheim University Hospital, Trondheim Norway
4. Department of Cancer Research and Molecular Medicine, Faculty of Medicine and Health Sciences, NTNU - Norwegian University of Science and Technology, Trondheim, Norway
5. Clinic of Surgery, St. Olav’s Hospital, Trondheim University Hospital, Trondheim, Norway

^Δ^ These authors contributed equally to this work

*Corresponding authors:

Elise Sandsmark: [elise.sandsmark@gmail.com](mailto:elise.sandsmark@gmail.com)

May-Britt Tessem: [may-britt.tessem@ntnu.no](mailto:may-britt.tessem@ntnu.no)

MR Centre, Department of Circulation and Medical Imaging, Faculty of Medicine and Health Sciences, Norwegian University of Science and Technology, P.O. Box 8905, N-7491 Trondheim, Norway

**Supplementary Table S1.** Results from t-test of *SFRP4* expression between low (≤T2c) and high pathological T-stage (≥T3a) and Pearson correlation between preoperative PSA and *SFRP4* expression in the gene expression cohorts. The Sboner et al. and Erho et al. cohorts lacked information about PSA and pathologicalT-stage.

| **Cohorts** | ***SFRP4* expression and pathological T-stage** | | ***SFRP4* expression and PSA** | |
| --- | --- | --- | --- | --- |
|  | **Log_2_ fold change** | **p-value** | **Pearson’s *r*** | **p-value** |
| Study cohort | 1.03 | 0.017 | -0.27 | 0.119 |
| TCGA-PRAD | 1.05 | <0.001 | -0.08 | 0.096 |
| CAM  Ross-Adams et al. | 0.52 | 0.006 | 0.14 | 0.146 |
| STK  Ross-Adams et al*.* | 0.63 | 0.010 | -0.16 | 0.125 |
| Wang et al. | 0.05 | 0.876 | 0.14 | 0.345 |
| Sboner et al. | - | - | - | - |
| Taylor et al. | 0.48 | 0.003 | 0.00 | 0.961 |
| Mortensen et al. | 2.27­ | 0.004 | -0.06 | 0.729 |
| Erho et al. | - | - | - | - |

**Supplementary Table S2.** Correlations between citrate and spermine concentrations and gene expression of the genes in the NCWP-EMT gene expression signature in our study cohort. All variables were log_2_ transformed before the analyses.

| **Gene** | **Citrate** | | **Spermine** | |
| --- | --- | --- | --- | --- |
|  | **Pearson’s *r*** | **p-value** | **Pearson’s *r*** | **p-value** |
| *SFRP4* | -0.533 | <0.001 | -0.494 | <0.001 |
| *FZD2* | -0.421 | <0.001 | -0.350 | <0.001 |
| *SFRP2* | -0.354 | <0.001 | -0.31 | 0.002 |
| *LEF1* | -0.35 | 0.002 | -0.296 | 0.004 |
| *PLCB2* | -0.343 | <0.001 | -0.265 | 0.01 |
| *CDH11* | -0.342 | <0.001 | -0.258 | 0.012 |
| *CDH2* | -0.339 | <0.001 | -0.236 | 0.021 |
| *SFRP1* | -0.324 | <0.001 | -0.343 | <0.001 |
| *FYN* | -0.297 | 0.003 | -0.246 | 0.008 |
| *VIM* | -0.281 | 0.006 | -0.222 | 0.03 |
| *NKD2* | -0.270 | 0.008 | -0.235 | 0.022 |
| *TCF4* | -0.265 | 0.009 | -0.225 | 0.028 |
| *MMP9* | -0.185 | 0.073 | -0.118 | 0.257 |
| *CDH3* | -0.071 | 0.495 | -0.155 | 0.133 |
| *WNT5A* | 0.051 | 0.627 | 0.067 | 0.521 |

**Supplementary Table S3.** Scoring of SFRP4 immunohistochemistry.

| **Staining Index (SI) = Intensity score * Proportion score** | | | | |
| --- | --- | --- | --- | --- |
| **Intensity** | **0** | **1** | **2** | **3** |
| Cytoplasmic staining Most common intensity | No detectable signal | Weak signal seen at intermediate to high magnification | Moderate signal seen at low to intermediate magnification | Strongest signal seen at low magnification |
| **Proportion** | **0** | **1** | **2** | **3** |
| Proportion of cancer cells with positive staining | <1% | 1-10 % | 11-50 % | >50 % |

**Supplementary Table S4.** SFRP4 immunohistochemistry evaluation, Gleason score/Grade Group, follow-up, and metabolite concentrations of the samples/patients in our IHC cohort.

|  | **Immunohistochemistry SFRP4** | | | **Gleason score (Grade Group)** | **Biochemical recurrence** | | **Metabolites  (mmol/kg wet weight)** | |
| --- | --- | --- | --- | --- | --- | --- | --- | --- |
| **Patient** | **Intensity** | **Percentage** | **Staining index** |  | **Status** | **Time (months)** | **Citrate** | **Spermine** |
| 1 | 1.00 | 2.00 | 2.00 | 3+4=7 (2) | 1 | 27.57 | 1.16 | 0.09 |
| 2 | 1.00 | 3.00 | 3.00 | 4+3=7 (3) | 1 | 14.59 | 2.51 | 0.54 |
| 3 | 2.00 | 3.00 | 6.00 | 3+4=7 (2) | 1 | 1.44 | 5.03 | 0.52 |
| 4 | 2.00 | 2.00 | 4.00 | 4+3=7 (3) | 1 | 32.43 | 7.98 | 0.62 |
| 5 | ND | ND | ND | 3+4=7 (2) | ND | ND | 4.04 | 0.44 |
| 6 | 1.00 | 2.00 | 2.00 | 3+4=7 (2) | ND | ND | 11.61 | 0.82 |
| 7 | 0.00 | 0.00 | 0.00 | 4+3=7 (3) | 0 | 28.30 | 1.95 | 0.37 |
| 8 | 0.00 | 0.00 | 0.00 | 3+4=7 (2) | 0 | 82.89 | 8.04 | 0.78 |
| 9 | 1.00 | 3.00 | 3.00 | 3+3=6 (1) | 0 | 82.95 | 2.97 | 0.37 |
| 10 | 3.00 | 2.00 | 6.00 | 4+5=9 (5) | 0 | 81.90 | 13.17 | 0.98 |
| 11 | 1.00 | 3.00 | 3.00 | 3+4=7 (2) | 0 | 84.30 | 7.11 | 0.57 |
| 12 | 0.00 | 0.00 | 0.00 | 4+3=7 (3) | 0 | 83.18 | 19.48 | 1.79 |
| 13 | 1.00 | 2.00 | 2.00 | 4+5=9 (5) | 1 | 16.03 | 1.25 | 0.13 |
| 14 | ND | ND | ND | 3+4=7 (3) | 0 | 82.23 | 11.17 | 1.11 |
| 15 | 0.00 | 0.00 | 0.00 | 3+4=7 (3) | 0 | 68.92 | 6.98 | 0.60 |
| 16 | 1.00 | 3.00 | 3.00 | 4+4=8 (4) | 1 | 31.31 | 5.24 | 0.43 |
| 17 | 1.00 | 2.00 | 2.00 | 3+3=6 (1) | 0 | 63.67 | 6.05 | 0.38 |
| 18 | ND | ND | ND | 3+3=6 (1) | 0 | 80.39 | 13.89 | 1.16 |
| 19 | 1.00 | 2.00 | 2.00 | 4+4=8 (4) | 1 | 7.44 | 4.69 | 0.30 |
| 20 | 2.00 | 3.00 | 6.00 | 3+4=7 (2) | 0 | 59.25 | 6.56 | 0.71 |
| 21 | 1.00 | 2.00 | 2.00 | 4+4=8 (4) | 1 | 3.21 | 2.22 | 0.24 |
| 22 | ND | ND | ND | 3+4=7 (2) | 0 | 72.00 | 5.33 | 0.62 |
| 23 | 1.00 | 1.00 | 1.00 | 3+4=7 (2) | 1 | 43.05 | 3.91 | 0.52 |
| 24 | 2.00 | 2.00 | 4.00 | 3+4=7 (2) | 0 | 71.21 | 3.88 | 0.37 |
| 25 | 1.00 | 2.00 | 2.00 | 4+3=7 (3) | 0 | 71.80 | 11.67 | 0.71 |
| 26 | 1.00 | 3.00 | 3.00 | 4+3=7 (3) | 0 | 73.77 | 6.52 | 0.65 |
| 27 | 1.00 | 2.00 | 2.00 | 3+3=6 (1) | 0 | 35.74 | 3.96 | 0.29 |
| 28 | 2.00 | 3.00 | 6.00 | 5+5=10 (5) | 1 | 1.15 | 4.15 | 0.77 |
| 29 | ND | ND | ND | 4+3=7 (3) | 0 | 71.84 | 16.26 | 2.09 |
| 30 | 1.00 | 3.00 | 3.00 | 3+4=7 (2) | ND | ND | 0.77 | 0.00 |
| 31 | 2.00 | 2.00 | 4.00 | 4+4=8 (4) | 1 | 53.11 | 2.22 | 0.38 |
| 32 | 1.00 | 2.00 | 2.00 | 5+4=9 (5) | 1 | 32.33 | 4.32 | 0.71 |
| 33 | ND | ND | ND | 3+3=6 (1) | 0 | 72.30 | 8.93 | 1.31 |
| 34 | 0.00 | 0.00 | 0.00 | 3+4=7 (1) | 1 | 1.61 | 2.85 | 0.13 |
| 35 | 1.00 | 2.00 | 2.00 | 3+4=7 (1) | 0 | 72.85 | 1.91 | 0.08 |
| 36 | 0.00 | 0.00 | 0.00 | 4+4=8 (4) | 1 | 1.28 | 1.17 | 0.12 |
| 37 | 2.00 | 3.00 | 6.00 | 4+5=9 (5) | 1 | 62.03 | 8.74 | 1.40 |
| 38 | 1.00 | 2.00 | 2.00 | 3+4=7 (1) | 0 | 70.07 | 2.06 | 0.52 |
| 39 | ND | ND | ND | 4+3=7 (3) | 0 | 64.07 | 8.25 | 0.81 |
| 40 | 1.00 | 1.00 | 1.00 | 4+3=7 (3) | 1 | 55.31 | 3.69 | 0.39 |
| Abbreviation: ND – no data/excluded. For immunohistochemistry evaluation, samples were excluded because of low tumour content. For biochemical recurrence data, patients were excluded due to lack of follow-up data. Gleason score/Grade Group represents the samples, not the patients. Metabolite data were quantified with LCModel. | | | | | | | | |

**Supplementary Table S5.** Overview of cohorts with gene expression data

| **Cohort** | **Access number** | **Gene expression method** | **Cancer samples** | **Normal samples** | **Follow-up Endpoint** |
| --- | --- | --- | --- | --- | --- |
| Study cohort | E-MTAB-1021 | Microarray, Illumina HT 12v4 | RP | Same patients | BCR or salvage treatment |
| TCGA-PRAD | TCGA PRAD | RNA Sequencing | RP | Same patients | BCR |
| CAM  Ross-Adams et al. | GSE70768 | Microarray, Illumina HT  12v4 | RP | Matched benign tissue | BCR or salvage treatment |
| STK  Ross-Adams et al*.* | GSE70769 | Microarray, Illumina HT  12v4 | RP | - | BCR or salvage treatment |
| Wang et al. | GSE8218 | Microarray, Affymetrix gene chips U133A | RP | 4 Autopsy, biopsies smaller. | BCR |
| Sboner et al. | GSE16560 | Microarray, Illumina DASL Assay | TURP from watchful waiting cohort | - | Prostate cancer-specific death |
| Taylor et al. | GSE21034 | Microarray, Affymetrix Human Exon 1.0 ST | RP | From RP of PCa patients | BCR |
| Mortensen et al. | GSE46602 | Microarray, Affymetrix U133 Plus 2.0 | RP | Surgical specimens of prostate from cystectomy of bladder cancer patients | BCR |
| Erho et al. | GSE46691 | Microarray, Affymetrix Human Exon 1.0 ST GeneChips | RP | - | Metastatic progression |
| Abbreviations: RP – Radical prostatectomy, BCR – biochemical recurrence, TURP – Transurethral resection of the prostate | | | | | |
